# Physical Effects, Safety and Feasibility of Prehabilitation in Patients Awaiting Orthotopic Liver Transplantation, a Systematic Review

**DOI:** 10.3389/ti.2022.10330

**Published:** 2022-09-08

**Authors:** Wesley D. Jetten, Rianne N. M. Hogenbirk, Nico L. U. Van Meeteren, Frans J. C. Cuperus, Joost M. Klaase, Renate De Jong

**Affiliations:** ^1^ Department of Anesthesiology, Erasmus University Medical Center, Erasmus University Rotterdam, Rotterdam, Netherlands; ^2^ Department of Surgery, Division of Hepatopancreatobiliary Surgery and Liver Transplantation, University Medical Center Groningen, University of Groningen, Groningen, Netherlands; ^3^ Top Sector Life Sciences and Health (Health∼Holland), The Hague, Netherlands; ^4^ Department of Gastroenterology and Hepatology, University Medical Center Groningen, University of Groningen, Groningen, Netherlands

**Keywords:** prehabilitation, orthotopic liver transplantation, physical exercise training, aerobic capacity, safety, feasibility

## Abstract

Prehabilitation improves surgical outcomes in patients undergoing surgery. However, patients preparing for orthotopic liver transplantation (OLT) are physically “frail” and suffer from comorbidities that generally hamper physical activity. This systematic review aims to evaluate the physical effects, safety and feasibility of prehabilitation in OLT candidates. Relevant articles were searched, in Embase, Web of Science, Cochrane, Medline and Google Scholar, to December 2021. Studies reporting on specified preoperative exercise programs, including adult OLT candidates with end-stage liver disease, with a model for end-stage liver disease (MELD) score ≥12 or Child-Pugh classification B/C, were included. This resulted in 563 potentially eligible studies, out of which eight were selected for inclusion, consisting of 1,094 patients (male sex 68%; mean age 51–61 years; mean MELD score 12-21). Six of the included studies were classified as low-quality by the GRADE system, and three studies had high risk for ineffectiveness of the training program according to the i-CONTENT tool. Significant improvement was observed in VO2 peak, 6-minute walking distance, hand grip strength, liver frailty index and quality of life. Feasibility ranged from an adherence of 38%–90% in unsupervised-to >94% in supervised programs. No serious adverse events were reported. In conclusion, prehabilitation in patients awaiting OLT appears to improve aerobic capacity, and seems feasible and safe. However, larger clinical trials are required to accurately examine the preoperative and postoperative effects of prehabilitation in this specific patient population.

## Graphical Abstract

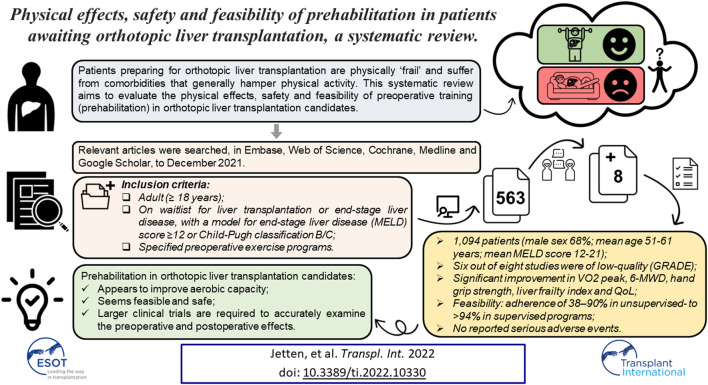

## Introduction

Poor physical fitness and functional status compromise postoperative functional recovery and lead to adverse postoperative outcomes, including complications, prolonged length of in-hospital stay, and mortality (1).

In current practice, patients who undergo (major) abdominal surgery are postoperatively supported by physical therapists and dieticians as part of the Enhanced Recovery After Surgery (ERAS^®^) program to accelerate postoperative recovery by enhancing perioperative health and reducing the impact of hospitalization and surgical stress (2,3). In addition, preoperative physical fitness measured by cardiopulmonary exercise tests has shown to be an independent predictor for postoperative morbidity and mortality after major abdominal surgery (4). Therefore, in the recent years, an increasing amount of scientific evidence focusses on preoperative “rehabilitation,” known as prehabilitation (5,6). Prehabilitation is aimed at strengthening the “psychophysiological reserve” and mitigating the postoperative surgical stress response to improve postoperative outcomes by enhancing preoperative general health and reducing individual risk factors (6).

Previous studies showed that prehabilitation programs are feasible, safe, and effective in patients scheduled for major abdominal surgery (7-9). However, the evidence regarding the beneficial effects of prehabilitation in patients awaiting orthotopic liver transplantation (OLT), a generally physically ‘frail’ patient population, is limited. OLT candidates not only exhibit key premorbid components of frailty, such as diminished functional capacity, sarcopenia, and decreased aerobic capacity, but may also suffer from cirrhosis-induced complications, such as ascites, hepatic encephalopathy, or variceal bleeding (10,11), which raises questions concerning their trainability. However, the waiting period for this procedure, on average 28 weeks in the Netherlands, 13–17 weeks in the United Kingdom, and 24 weeks in the United States of America, might be used to optimize physical condition by training prior to OLT (12-14).

Moreover, previous research in OLT candidates predicted a higher survival after OLT in patients with a higher anaerobic threshold (a submaximal exercise parameter of cardiorespiratory reserve) (15). Therefore, prehabilitation could possibly benefit patients in reducing morbidity and mortality during the waiting period or after OLT.

The primary objective of this systematic review is to evaluate the observed effects of preoperative training on physical and functional capacity, and to evaluate the effect of prehabilitation on postoperative surgical outcomes after OLT. The secondary objective is to determine the feasibility and safety of prehabilitation programs in patients awaiting OLT. In addition to the primary and secondary objectives, we aim to provide an overview of the studied prehabilitation programs, including their content and potential for success (16,17).

## Materials and Methods

### Study Design

This systematic review was conducted and reported in accordance with the Preferred Reporting Items for Systematic Reviews and Meta-Analyses (PRISMA) Statement (18,19), see Online Supplementary S1. Two authors (WJ, RH) independently reviewed the selected studies in EndNote X9© (Clarivate Analytics, Boston, MA, United States). Identified articles were screened on title, abstract, and, subsequently, on full-text. Disagreements during the selection process were discussed by the two reviewing authors (WJ and RH) and a third author (RJ) until consensus was reached.

### Search Strategy

The search strategy was developed in collaboration with a clinical librarian and information specialist and was executed in Embase, Web of Science, Cochrane, Medline (PubMed) and Google Scholar. Free text words and MeSH terms related to prehabilitation and liver transplantation were used. Reference lists of relevant review articles and current treatment guidelines were screened for additional eligible articles. All studies published before 21 December 2021 were included for screening by title and abstract. The full literature database search strategy is described in Online Supplementary S2 *.*

### Eligibility Criteria

All peer-reviewed randomized, controlled, and cohort studies reporting a specified preoperative exercise program for adult (age ≥18 years) patients actively listed for OLT or with end-stage liver disease (ESLD). To assess ESLD, the model for end-stage liver disease (MELD) score, a disease severity scoring system used to improve organ allocation for patients on the liver transplantation waiting list, and the Child-Pugh classification were used. Studies that assessed patients with a laboratory or exception MELD score ≥12 or a Child-Pugh classification B or C were included. Animal studies, case-reports, systematic reviews, conference abstracts, duplicates and studies containing paediatric patients were excluded.

### Quality Assessment

Quality assessment of included studies was executed by using the principles of the Grading of Recommendation, Assessment, Development, and Evaluation (GRADE) (20,21). For a transparent assessment of the potential effectiveness of the exercise therapy programs studied in trials, intervention programs were evaluated according to the international Consensus on Therapeutic Exercise Training (i-CONTENT) tool (17). The i-CONTENT is used to assess the therapeutic quality of exercise programs employed in clinical trials (17).

### Primary and Secondary Outcomes

The primary outcome was defined as the observed effects of preoperative training programs on physical and functional capacity and surgical outcome. Physical and functional capacity was assessed by comparing outcomes such as pre- and post-training oxygen consumption at peak exercise (VO2-peak), 6-minute walking distance (6MWD), hand grip strength, and quality of life (QoL). Surgical outcome was assessed by comparing data on post-OLT complications, length of in-hospital stay, length of intensive care unit (ICU) stay, and mortality.

Secondary outcomes were safety and feasibility of study- and training programs. The safety of training programs was assessed by comparing the occurrence and types of serious adverse outcomes during the training. The feasibility of studies was assessed by comparing patients identified as eligible for inclusion with the total number of included patients. The feasibility of training programs was assessed by an evaluation of the adherence to the training programs during the waiting period prior to OLT.

### Data Collection and Definitions

Following the screening and selection of included studies, data was extracted by two independent authors (WJ, RH). Patient characteristics extracted included age; sex; body mass index (BMI); (lab and/or exception) MELD score; Child-Pugh classification and comorbidities, including diabetes mellitus, cardiac disease, pulmonary disease, ascites, gastroesophageal varices, and hepatic encephalopathy. Data regarding primary and secondary outcomes were extracted and tabulated. In addition, rationales, designs of the training programs, data on duration, frequency of training and exercises, training intensity and context, supervision of the training programs, and their potential for success were tabulated to provide a detailed overview of the prehabilitation programs. Normally distributed variables are presented as means with standard deviation (SD) and skewed variables as medians with interquartile range (IQR).

## Results

### Search Results

**FIGURE 1:**
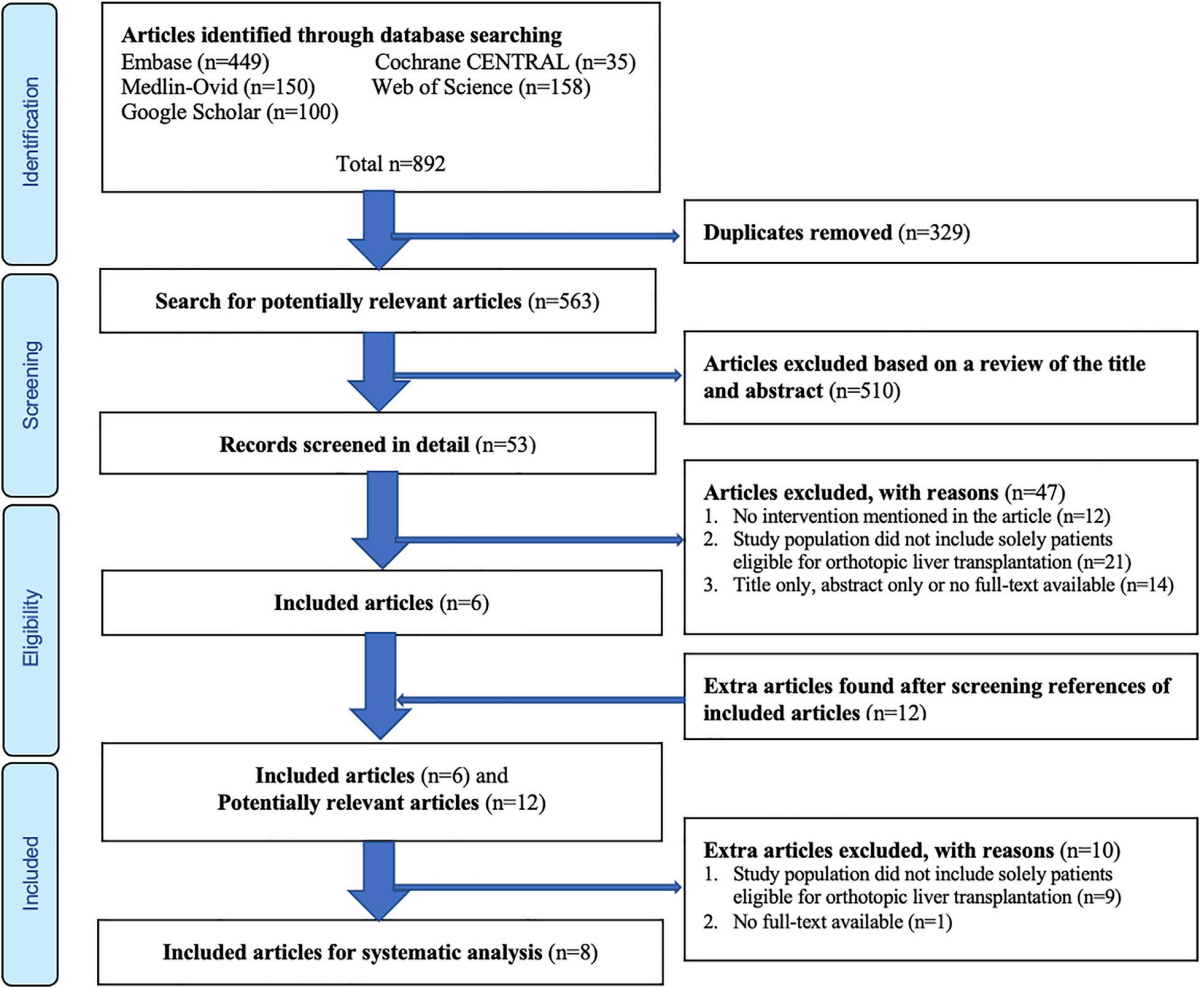
Flow diagram of the article selection procedure based on the Preferred Reporting Items for Systematic Reviews and Meta-Analyses (PRISMA) guideline.

The search of aforementioned databases provided a total of 892 articles possible for inclusion. After removing duplicates, 563 articles remained for screening by title and abstract. Of these, 510 were excluded based on titles and abstracts. The full-texts of the remaining 53 articles were assessed for eligibility and reviewed in detail, whereafter 47 papers were excluded and six papers were included (Figure 1). Eventually, another 12 potentially relevant articles were found by screening references from articles that were already included for analysis. Of this total of 18 remaining articles, another 10 were excluded (Figure 1), and eight full-text studies (11,22-28) remained for systematic analysis (Table 1).

**TABLE 1:** Designs of included studies and patient demographics.

Author	Limongi (22)	Debette-Gratien (23)	Al-Judaibi (11)	Wallen (24)
Year	2014	2015	2019	2019
Study Design	Randomized controlled trial	Prospective cohort study	Retrospective cohort study	Randomized controlled trial
Study quality^a^	Low	Low	Low	Moderate
Population (n)				
Training group	5	13	258	11
Control group	12	NA	200	10
Demographics				
Age, years				49 (40–60)^b^
Training group	53.41 (8.42)	51 (12)	53.4 (9.6)	NR
Control group	56.2 (3.96)	NA	56.5 (10.7)	NR
Sex, male, %				81%
Training group	92%	77%	26%	NR
Control group	60%	NA	68%	NR
BMI, kg/m^2^				
Training group	31 (7.4)	NR	NR	NR
Control group	28 (3.8)	NA	NR	NR
MELD-score				13.3 (4)
Training group	17.58 (4.46)	13 (6)	18 (6–40)^c^	NR
Control group	17 (3.93)	NA	21 (4–40)^c^	NR
Child Pugh-score				63%^d^
Training group	NR	B7 (3)	NR	NR
Control group	NR	NA	NR	NR
Comorbidities, n(%)				
Diabetes Mellitus				33%
Training group	3 (60%)	NR	90 (35.9%)	NR
Control group	3 (25%)	NA	43 (21.5%)	NR
Cardiac disease				0%
Training group	1 (20%)	0 (0%)	27 (10.8%)	NR
Control group	0 (0%)	NA	2 (1%)	NR
Pulmonary disease				
Training group	1 (20%)	NR	NR	NR
Control group	2 (17%)	NA	NR	NR
Ascites				
Training group	2 (40%)	2 (15%)	NR	NR
Control group	8 (67%)	NA	NR	NR
Gastroesophageal Varices				81%
Training group	NR	NR	NR	NR
Control group	NR	NA	NR	NR
Hepatic encephalopathy				
Training group	NR	NR	NR	NR
Control group	NR	NA	NR	NR

Author	Williams (25)	Morkane (26)	Chen (27)	Lin (28)
Year	2019	2019	2020	2021
Study Design	Prospective cohort study	Prospective cohort study	Randomized controlled trial	Ambispective cohort study
Study quality^a^	Low	Low	Low	Moderate
Population (n)				
Training group	18	16	9	517
Control group	NA	17	8	NA
Demographics				
Age, years				
Training group	55 (44-63)^b^	55.6 (7.8)	55 (7)	61 (53-66)^b^
Control group	NA	55.6 (7.8)	54 (11)	NA
Sex, male, %				
Training group	50%	88%	56%	59%
Control group	NA	82%	75%	NA
BMI				
Training group	25.4 (21-45) ^b^	30.9 (5.6)	31 (8)	30 (25-34) ^b^
Control group	NA	27 (4.7)	29 (4)	NA
MELD-score				
Training group	13 (12-26)^2^	13.7 (4.6)	16 (4)	12 (8-16) ^b^
Control group	NA	13.2 (3.7)	19 (3)	NA
Child Pugh-score				
Training group	NR	NR	9 (100%)^d^	NR
Control group	NR	NR	8 (100%)^d^	NR
Comorbidities, n (%)				
Diabetes Mellitus				
Training group	1 (5.6%)	NR	4 (45%)	227 (44%)
Control group	NA	NR	1 (13%)	NA
Cardiac disease				
Training group	1 (5.6%)	NR	NR	57 (11%)
Control group	NA	NR	NR	NA
Pulmonary disease				
Training group	NR	NR	NR	36 (7%)
Control group	NR	NR	NR	NA
Ascites				
Training group	6 (33%)	NR	7 (78%)	352 (69%)
Control group	NA	NR	6 (75%)	NA
Gastroesophageal Varices				
Training group	NR	NR	5 (56%)	340 (67%)
Control group	NR	NR	7 (88%)	NA
Hepatic encephalopathy				
Training group	6 (33.3%)	NR	9 (100%)	271 (53%)
Control group	NA	NR	8 (100%)	NA

a Quality assessment according to the GRADE system for quality assessment of clinical studies (20).

b Data presented as median (IQR).

c Data presented as median (range).

d no of patients with Child Pugh B or C.

Data are presented as mean (SD) unless stated otherwise.

BMI, body mass index; MELD, model for end-stage liver disease score; NA, not applicable; NR, not reported.

### Methodological Quality of Evidence Assessment

According to the GRADE system (20), two studies (24,28) were classified as moderate, while six (11,22-27) were classified as low-quality evidence, mainly due to the risk of bias and imprecision (Table 1). According to the i-CONTENT tool (17), five studies (23-28) were classified as low risk and three (11,22,27) as high risk for ineffectiveness of the training program. The main reason for high risk of ineffectiveness was due to unsupervised training (22,25,27,28) and missing reports on exercise-related adverse events (11,22,27,28) and adherence to the exercise program (11,22,23,27) (Table 2). For a detailed description of the grading process with the GRADE system and i-CONTENT tool*,* see Online Supplementary S3, S4 *,* respectively*.*

**TABLE 2:** Details of included training programs.

Author	Limongi (22)	Debette-Gratien (23)	Al-Judaibi (11)	Wallen (24)
Exclusion criteria	Age <18; Acute liver failure	No prevention of esophageal bleeding (β-blockers or varices ligation); Ventricular ejection fraction <45%; Arrhythmia/cardiac decompensation during excercise	None	Previous LT; Listed for other organ transplantation; Current smoking; Adverse event during CPET; Uncontrolled diabetes; Orthopedic/neurological limitation to exercise
Training details				
Training group	Physical training	Physical training	Physical training and nutritional support	Physical training
Control group	No exercises.	NA	Before implementation of training program.	No information regarding exercise training or physical activity provided.
Supervision training	Unsupervised training at home by manual.	Supervised in-hospital gym.	Supervised in hospital gym or unsupervised at home with twice/thrice weekly supervision through phone calls	Supervised in hospital gym and unsupervised at home.
Duration training, weeks	12	12	Until suitable for transplantation	8
			Mean duration not reported	
Frequency training	Daily	Twice weekly	1-5 times weekly	Thrice weekly
Type of training	1. Cough and breathing exercises	1. Aerobic training (cycle ergometer)	1. Aerobic training (cycle ergometer)	1. Aerobic training (cycle ergometer or walking)
	2. Isometric force exercises.	2. Muscle strength exercise (Press body building type)	2. Resistance strength exercise	2. Resistance strength exercise (circuit-based with weights)
			3. Education regarding activity.	
Risk of ineffectiveness of training program^a^	High	Low	High	Low

Author	Williams (25)	Morkane (26)	Chen (27)	Lin (28)
Exclusion criteria	Cardiovascular instability; CVA; ≥ grade 2 hepatic encephalopathy	Noncirrhotic liver disease; oncological diagnosis; contraindication for exercise	Large gastrointestinal varices without β-blocker use; HCC; hepatic encephalopathy; hydrothorax; pulmonary vascular complications of portal hypertension; cardiorespiratory contraindications for exercise	No exclusion criteria
Training details				
Training group	Physical training	Physical training	Physical training and nutritional support	Exercise prescription and one dietary consultation
Control group	NA	CPET at 0, 6 and 12 weeks, no exercise program	Nutritional support only	NA
Supervision training	Unsupervised at home	Supervised in hospital gym	Unsupervised training at home	Unsupervised home-based exercise workouts
	Once weekly supervision through phone calls		Weekly supervised counseling and daily motivational phone calls	Rarely: supervised home-based or outpatient physical therapy
				Once monthly phone follow-up and appointment after 90 - 120 days
Duration training (weeks)	12	6	12	Until LT
Frequency training	Twice weekly, 20 minutes exercise	Thrice weekly, 40 minutes.	Recommendation of 5 times weekly, 30 minutes.	Recommendation of 5 times weekly, 30 minutes
	Thrice daily, 10 minutes walking.			
Type of training	1. Functional resistance exercises (video guide)	Aerobic training (cycle ergometer)	Walking training by increasing daily step-goal (Fitbit).	Home exercise program:
	2. Aerobic exercises (video guide)			1. force: weights / resistance bands
	3. Walking program (daily step targets)			2. aerobic: treadmills, elliptical or stationary bikes
Risk of ineffectiveness of training program^a^	Low	Low	High	Low

a Risk of ineffectiveness of training program according to the i-CONTENT tool for assessing therapeutic quality of exercise programs employed in clinical trials (17)

LT, liver transplantation; CPET, cardiopulmonary exercise test; NA, not applicable.

### Included Studies

Eight studies investigating a total of 1,094 patients (median (IQR): 20 (17–139)) were included. A total of three randomized controlled trials (RCTs) (22,24,27), one ambispective cohort study (28), three prospective cohort studies (23,25,26), and one retrospective cohort study (11) were included. The contexts of the training programs varied between supervised in-hospital training (11,23,24,26) and unsupervised home-based training (11,22,24,25,27,28).

### Demographics, Primary and Secondary Outcomes

The majority of patients were male (68%). The mean or median age of the patients included in the training programs and control groups ranged from 51 to 61 and 54 to 56, respectively. In the studies reporting BMI, mean BMI in the training groups was ranging from 25.4 to 31 (22,25-28), which was higher than in the control groups (range 27–29) (22,26,27). The mean and median MELD-scores differed between studies, with five studies reporting mean or median MELD-scores between 12 and 14 in the training group (23-26,28), while, in the other three studies, these scores were above 16 in both the intervention and control groups (11,22,27). Six studies reported on the presence of one or more cirrhosis-induced comorbidities as ascites, gastroesophageal varices and hepatic encephalopathy (22-25,27,28). The number of patients with ascites ranged from 15% to 78% in the training groups (22-25,27,28) compared to 67%–75% in the control groups (22,27). The reported prevalence of hepatic encephalopathy ranged from 33% to 100% in the training groups (25,27,28) and 100% in the control groups (27). Three studies reported a prevalence of gastro-oesophageal varices ranging from 56% to 81% in the training group (24,27,28) and 88% in the control group (27). Baseline study characteristics and demographics are displayed in Table 1.

The primary outcomes reported on in the included studies varied and included alterations in spirometry results (22), alterations in frailty metrics (28), readmissions within 90 days post-OLT (11), and the safety and feasibility of training (23-25). The secondary outcomes were more uniform between the studies and included general QoL assessments (22-24,27), aerobic functioning after training (23-28), and adverse events during the program (11,22-27).

### Intervention

Three of six studies that implemented unsupervised home-based training programs provided once-to-thrice weekly telephone contact for supervision or motivational support (11,25,27). The duration of training programs varied from six (26) to eight (24) to 12 weeks (22,23,25,27) and until OLT (28). The frequency of training varied per study; Limongi et al. provided a manual for daily, non-supervised, home-based exercise training (22), while Debette-Gratien et al. implemented twice-weekly, supervised, in-hospital gym training (23). Thrice weekly supervised in-hospital training was utilized by Wallen, Morkane, and Al-Judaibi et al. (11,24,26), Williams, Chen and Lin et al. advised non-supervised training up to five times per week, dependent on pre-defined weekly targets (25,27,28).

Physical training programs mainly consisted of aerobic training by cycle ergometer or walking programs (11,22-28), and strength exercises (11,22-25,28), or coughing and breathing exercises (22). Except for the interval training described by Morkane et al., Debette-Gratien et al. and Williams’ set goal to archive a work rate of 12–14 on the Borg scale of rate of perceived exertion (RPE-score) (23,25,26,29), no insight was provided into the intensity of the training programs in the other included studies. Al-Judaibi and Lin et al. provided education related to physical activity and dietary support in the training group (11,28), whereas, in Chen et al.’s study, both the intervention and control groups were provided with extra information regarding nutrition (27). Detailed information regarding the designs of the training programs, exclusion criteria, supervision, duration, and the risk of ineffectiveness is provided in Table 2.

### Data-Analysis

#### Effect of Training on Physical Capacity

All the studies examining the physical effects of aerobic training reported some significant improvement in aerobic capacity (23-28) (Table 3). Debette-Gratien et al. reported a significant improvement in VO2 peak after training, from a mean VO2 peak of 21.5 (5.9) ml/kg/min before training to a mean VO2 peak value of 23.2 (5.9) ml/kg/min after training (*p* = 0.008) (23). In addition, Morkane et al. reported a significant VO2 peak improvement of 2.3 ml/kg/min in the training group (*p* = 0.02), while a decrease of 1.9 ml/kg/min was observed in the control group (p = 0.03) (26). Although Chen et al. found no significant improvement in VO2 peak after training (18 (7) before versus 17 (6) ml/kg/min after training, *p* = 0.42), they observed a decrease of 3 ml/kg/min (*p* = 0.08) in the control group (27). Debette-Gratien, Wallen, and Chen et al. reported significant improvements in walking distance after training (+40 m, *p* = 0.02; +16 m, p = 0.02 and +59m, p = 0.05, respectively) (23,24,27), while Lin et al. did not report a significant improvement in walking distance after training (F = 2.64, *p* = 0.07) (28). Furthermore, Debette-Gratien and Morkane et al. reported a significant improvement in grip strength (+7 kg, *p* = 0.008 and +3 kg, *p* = 0.05, respectively) after 12 weeks of training (23,26). However, in the study of Wallen et al., there was no significant improvement in grip strength after training (+0.4 kg, *p* = 0.24) (24). Regarding 6MWD and hand grip strength, no significant improvement or decline was observed in the control groups. Although Williams et al. did not report on VO2 peak or 6MWD, they did observe a significant improvement in aerobic capacity, measured by the incremental shuttle walk test (ISWT) (260 (70–1020) meter to 470 (190–880) meter, p < 0.01), and functional capacity, measured by the Short Physical Performance Battery Test (SPPBT) (9.5 (6–12) to 11.5 (9–12), p = 0.02), after 12 weeks of training. Lin et al. found a significant improvement of the liver frailty index (LFI) for all patients after training (F = 3.45, p = 0.01), and found an even larger effect in patients who adhered to >80% of the workout sessions until OLT (F = 8.10; p < 0.001) (28). Thereby, Lin et al. found a significant correlation with an improvement of the LFI and a survival advantages among included patients (28).

**TABLE 3:** Physical effects of training in patients awaiting orthotopic liver transplantation.

Author	Aerobic capacity						Functional capacity		
	VO2 peak (ml/kg/min)			6MWD (m)			Handgrip strength (kg)		
	Before training	After training^a^	*p*-value	Before training	After training^a^	*p*-value	Before training	After training^a^	*p*-value
Debette-Gratien (23)	21.5 (5.9)	23.2 (5.9)	**0.008**	481 (69)	521 (64)	**0.02**	30 (10)	37 (13)	**0.008**
Wallen (24)									
Training/control^b^	NR	NR		NR	+103.8 (81.4)	**0.02**	NR	+6.3 (8.5)	0.24
Morkane (26)									
Training	16.2 (3.4)	18.5 (4.6)	**0.02**	NR	NR		26.4 (7.5)	29.4 (6.4)	**0.05**
Control	19.0 (6.1)	17.1 (6.0)	**0.03**	NR	NR		29.1 (10.7)	30.5 (13)	0.8
Chen (27)									
Training	18 (7)	17 (6)	0.42	423 (60)	482 (87)	**0.05**	NR	NR	
Control	18 (6)	15 (7)	0.08	418 (59)	327 (166)	0.21	NR	NR	
	**GST (m/s)**						**LFI**		
Lin (28) ^c^	Before training	After training	*p*-value	Before training	After training	*p*-value	Before training	After training	*p*-value
Training (all patients)	1.0 (0.8–1.2)	*F* = 1.53	0.20	326 (244–390)	*F* = 1.88	0.13	3.8 (3.3–4.5)	*F* = 3.45	**0.01**
Training (full adherence group)^d^	1.0 (0.8–1.2)	*F* = 1.20	0.32	326 (244–390)	*F* = 2.64	0.07	3.8 (3.3–4.5)	*F* = 8.10	**<0.001**
Control	NR	NR	NR	NR	NR	NR	NR	NR	NR
				**ISWT (m)**			**SPPBT**		
				Before training	After 12 weeks	*p*-value	Before training	After 6 weeks	*p*-value
Williams (25)	NR	NR	NR	260 (70–1020)	470 (190–880)	**<0.01**	9.5 (6-12)	11.5 (9-12)	**0.02**
	**FVC (%)**			**FEV1 (%)**					
	Before training	After training^a^	p-value	Before training	After training^a^	p-value			
Limongi (22)									
Training	82.8 (13.1)	87 (7.9)	NR	76 (17)	82 (14.5)	NR	NR	NR	
Control	84.3 (12.2)	87 (19.2)	NR	84.3 (12.8)	85.4 (15.2)	NR	NR	NR	
Al-Judaibi (11)	NR	NR		NR	NR		NR	NR	

a The control group did not receive any training.

b Only between-group changes (intervention vs. control) were reported in the study.

c This study did not mention after-training outcomes as absolute numbers, but as delta points (F).

d Full adherence: study patients who completed >80% of workout sessions.

Data are presented as mean (SD) or median (IQR).

VO2 peak, oxygen consumption at peak exercise; 6MWD, 6-minute walking distance; F, delta points; GST, gait speed test; LFI, liver frailty index; FVC, forced vital capacity; FEV_1_ = forced expiratory volume in one second; ISWT, incremental shuttle walk test; SPPBT, short physical performance battery test; NR, not reported.

#### Perceived Health-Related Quality of Life Before and After Training

Four studies examined QoL before and after the training program while awaiting OLT (Table 4) (23-25,27). Williams et al. found an increase of 18% reported in the EuroQol visual analogue scale (EQ-VAS) questionnaire (*p* = 0.04) (25,30). And, although Williams et al. found no differences in median hospital anxiety and depression score (HADS) (10 (1–26) before training versus 7 (0–22) after training, *p* = 0.13), an increase of proportion of patients reporting no problems with mobility (44%) and pain/discomfort (56%) in the EuroQol 5-Dimension 5-Level (EQ-5D-5L) instrument was found after 12 weeks of prehabilitation (25). Debette-Gratien, Wallen, and Chen et al. found no differences in QoL between the training and control groups or between pre- and post-training on the SF-36 (24) or the HR-QoL (23,24,27). However, in Chen et al.’s study, an improvement was observed on the sickness impact profile (SIP) in the training group (−4.2, *p* = 0.10), while the SIP in the control group worsened (+4.2, *p* = 0.07) (27).

**TABLE 4:** Effect of training on quality of life in patients awaiting orthotopic liver transplantation.

Author	Tool	Quality of life		
		Before training	After training^a^	*p*-value
Debette-Gratien (23)	SF-36	36 (4)	39 (3)	0.46
Wallen (24)				
Training/control^b^	HR-QoL	NR	−0.3 (−1.3,0.8)	0.67
Williams (25)	EQ-VAS	NR	“Improvement of 18%”	**0.04**
	EQ-5D	NR	Improvement in: 44% - Mobility	
	No-problems reported		56% - Pain/discomfort	
	HADS	10 (1–26)	7 (0–22)	0.13
Chen (27)				
Training	SIP	11.2 (7.3)	7 (6.4)	0.10
Control^a^	SIP	11.5 (13)	15.7 (17.3)	0.07
Limongi (22)	NR			
Al-Judaibi (11)	NR			
Morkane (26)	NR			
Lin (28)	NR			

a The control group did not receive any training.

b Only between-group changes (intervention vs. control) were reported in the study.

Data are presented as mean (SD) or median (IQR).

SF-36, Short Form 36; HR-QoL, health related quality of life; EQ-VAS, EuroQol visual analogue scale; EQ-5D, european quality of life five dimensions; HADS, hospital anxiety and depression score; SIP, sickness impact profile; NR, not reported.

#### Effects of Training on Length of Hospital Stay After OLT

Two studies (11,26) described differences in the length of in-hospital stay after OLT between the training groups and control groups (Table 5). Al-Judaibi et al. found a significantly shorter median length of ICU stay before transplantation in the intervention group compared to the control group (n = 458, 2 vs. 3 days, *p* = 0.01), however, no significant difference was observed in the length of in-hospital stay after OLT (11). Morkane et al. found no difference in the median length of ICU stay between the intervention and control groups (2 (4) versus 4 (5.5), *p* = 0.77), but found a significant difference in postoperative median length of hospital stay between the training group and control group (13 (7–19) versus 30 (17–43), *p* = 0.02) (26).

**TABLE 5:** Effect of training on postoperative surgical outcome after orthotopic liver transplantation.

Author	Length of hospital stay (days)	*p-value*	Length of ICU stay (days)	*p-value*	90-day readmission rate	*p-value*
Williams (25)	10 (5–41)		4 (1)		NR	
Al-Judaibi (11)						
Training	14 (3–150)	0.69	NR		17%	0.58
Control	17 (5–161)		NR		20%	
Morkane (26)						
Training	13 (7–19)	**0.02**	2 (4)	0.77	NR	
Control	30 (17–43)		4 (5.5)		NR	
Debette-Gratien (23)	NR		NR		NR	
Limongi (22)	NR		NR		NR	
Wallen (24)	NR		NR		NR	
Chen (27)	NR		NR		NR	
Lin (28)	NR		NR		NR	

Data are presented as mean (SD), median (IQR) or n (%).

ICU, intensive care unit; NR, not reported.

#### Feasibility of the Studies Performed

Three studies reported on the participants identified for possible inclusion and the reasons for exclusion. Wallen et al. identified 38 patients, of whom 15 declined to participate; one patient was transplanted before the start of the training program, and another was delisted before commencement, leaving 21 (55%) suitable for inclusion (24). Chen et al. identified 227 OLT candidates and excluded 210 (93%) for various reasons: 85 because of the presence of a hepatocellular carcinoma, 73 due to logistic or transport issues, 35 because of cardiopulmonary or metabolic diseases, 14 because of being delisted as OLT candidates, two due to repeated hospitalization, and one because that patient already walked more than 10,000 steps per day (27). Williams et al. randomly selected 46 patients from the OLT waiting list: 32 (70%) were eligible for study entry, with patients awaiting a re-transplantation being the most common reason for exclusion (5 out of 46; 11%). Of the 32 patients deemed eligible, six (18.8%) declined participation and eight (25%) underwent OLT prior to study visit one. Therefore, a total 18 out of 32 eligible patients (56.2%) were enrolled in the study (25). Al-Judaibi et al. Debette-Gratien et al. and Lin et al. included consecutive patients and had a study feasibility of 100% (11,23,28). Limongi et al. identified 42 patients and included 17 (40%) in their study without listing reasons for exclusion (22), and Morkane et al. did not report on patients eligible for inclusion (26). No studies excluded patients with gastro-oesophageal varices treated with β-blockers (Table 2).

#### Feasibility and Safety of Training Programs

Outcomes regarding safety, feasibility, and adherence to the training programs are displayed in Table 6. Three author groups reported the feasibility and safety of their training programs as their primary outcome (24-26). Williams et al. defined feasibility as the absence of training-related serious adverse events; the eligibility of 66% or more of patients who are actively listed on the OLT waiting list; and more than 66% adherence to the daily step count and resistance exercises and completion of 6 weeks training (25). In their study, 82% of the patients adhered to daily step targets and 90% to the twice-weekly exercises (25). Morkane et al. reported a 94% adherence with all exercises (26), and Wallen et al. reported a 95% and 75% adherence to supervised and unsupervised exercise training, respectively (24). Lin et al. reported an adherence to minimally one follow-up physical therapy session of 69% (28). Patients’ self-reported adherence varied from adherence of 4–5 days/week in 38% of the patients, to 1–3 days/week in 51% of the patients and 0 days/week in 11% of the patients (28).

**TABLE 6:** Feasibility and safety of prehabilitation in patients awaiting orthotopic liver transplantation.

Author	Feasibility/Adherence to the program	Safety and adverse events	No. patients lost to follow up intervention group
Debette-Gratien (23)	NR	1 – worsening hepatorenal syndrome	2 – moved to another region
		No cardiovascular events	2 –transplanted before 12 weeks
		No cirrhotic decompensation	1 – deterioration of clinical condition
		No variceal bleeding or ascites)	
Wallen (24)	95% adherence to supervised exercise training	1 – adverse event (knee injury)	5 – transplanted before 8 weeks
	75% adherence to unsupervised exercise training	No serious adverse events	1 – delisted and noncompliant
		No variceal bleeding or hepatic encephalopathy	
Williams (25)	82% adherence to step-targets	No adverse events	1 – non-study related trauma
	90% adherence to twice weekly exercises		
Morkane (26)	94% of total exercise sessions were completed	No adverse events	1 – transplanted before 12 weeks
		No worsening cirrhotic decompensation	
Chen (27)	NR	NR	1 – other surgery
			1 – transplanted before 12 weeks
			1 – lost to follow-up
Lin (28)	Adherence to minimally 1 follow up:	NR	24 – failed to visit follow up sessions; unknown reason
	211 (69%) of 305 LT-candidates		
	Self-reported adherence:		
	4–5 day/week: 146 (38%)		
	1–3 day/week: 198 (51%)		
	0 days/week: 41 (11%)		
Limongi (22)	NR	NR	NR
Al-Judaibi (11)	NR	NR	NR

LT, liver transplantation; NR, not reported

Four studies (23-26) described the potential of serious adverse events resembling cardiovascular events, cirrhosis decompensation, variceal bleeding or hepatic encephalopathy, but none of the authors reported any of these events occurring during the study. Wallen et al. reported on one adverse event (knee injury, one out of 11 patients (9.1%)) that occurred during training (24). In the study of Debette-Gratien et al., one patient (one out of 13 patients (7.7%)) stopped training due to worsening of their hepatorenal syndrome (23). Most common reason for dropping out of the program was because of transplantation before the end of the study period. All reasons why patients were lost to follow-up are listed in Table 6.

## Discussion

The aim of this systematic review was to evaluate the effect of prehabilitation on physical capacity and surgical outcome in patients actively waiting for OLT. Six out of eight studies demonstrated significant improvements in aerobic or physical capacity (23-28). Adherence to the training programs was 69% or higher, and none of the included studies reported any serious adverse events. Therefore, these findings imply that prehabilitation programs are safe, feasible, and, potentially, effective for OLT-candidates.

In the past, one other review and one meta-analysis have been conducted in patients with chronic liver disease to assess the effect of training on their physical capacity (31,32). And although this current review shows resemblance to these previously conducted reviews, the majority of their included studies excluded potential OLT candidates and patients with MELD score ≥12, while this current review solely focussed on patients with ESLD awaiting OLT (31,32). For example, in the review conducted by Williams et al., the authors concluded that moderate‐to-high intensity exercise can improve the physical components of frailty and QoL in patients with chronic liver disease, but that it remained to be elucidated whether this also applies to patients with Child Pugh B/C decompensated cirrhosis (33-39). In the review of Brustia et al., where not solely patients awaiting OLT were included, no adverse events were caused by the training, but neither an improvement in physical capacity was observed (32).

When elaborating on the physical effects of prehabilitation in OLT candidates, previous literature has shown that preoperative VO2 peak and MELD score are independent prognostic factors of mortality and duration of hospitalization during both the pre- and post-transplantation periods (15,40-42). Hence, it can be hypothesized that increased VO2 peak due to training, could improve surgical outcome for the OLT candidate. The ability to increase this physical capacity with training was shown by several studies included in this review (23-27). The studies of Debette-Gratien, Morkane and Williams et al. all found a significant improvement in aerobic capacity after training (23,25,26). Their results, however, differed from the study by Chen et al., who found no difference in VO2 peak after training (27). This difference in results might be explained by the differences in design of the training programs of the three studies: Debette-Gratien and Morkane et al. provided specified supervised aerobic training with a cycle ergometer, Williams et al. used video guided exercises and non-supervised walking training, and Chen et al. solely implemented non-supervised walking training (23,25-27). Thereby, only three out of eight studies outlined the aerobic intensity of the exercises (23,25,26). Debette-Gratien et al. and Morkane et al. based their patient-adjusted aerobic training protocol on VO_2_ peak and on the anaerobic threshold which was objectified by CPET (23,26). Williams et al. used a subjective scale where patients were asked to achieve a work rate of 12–14 on the Borg scale (25). To speculate, these results should be interpreted with caution, but suggest that supervised aerobic cycling training by use of a patient-adjusted protocol could be more beneficial than unsupervised walking training. The hypothesis that physical training improves postoperative recovery was only described in three out of the eight included studies (11,25,26), and seems to be consistent with the findings of Lin et al., who found a significant correlation between survival advantage with improvement of the LFI score (28), and Morkane et al., who found a significant median difference of 17 days in the length of in-hospital stay between the intervention and control groups (26). However, in contrast with Morkane et al.’s finding, Al-Judaibi et al. found no difference in the length of in-hospital stay or 90-day readmission rate (11). The differences between the studies of Morkane and Al-Judaibi et al. may be explained by the studies’ population sizes (*n* = 17 vs. *n* = 458, respectively) and the significantly older population with more comorbidities in the training group compared to the control group in the study of Al-Judaibi et al. (11), while in the study of Morkane et al. no significant differences in baseline demographics of the two groups were reported (26).

Debette-Gratien et al. were able to include 100% of eligible candidates in their study (23), while Chen et al. only included 7% of eligible candidates. This discrepancy between eligible and eventually included patients could be caused by tight inclusion criteria, but results in a questionable feasibility of the study and could increase the risk of potential attrition bias. However, when evaluating feasibility of the training programs, all studies that mentioned adherence to the program reported a 38–90% adherence in unsupervised exercise training (24,25,28) and 94% or higher adherence to supervised exercise training (24,26), suggesting a high feasibility of prehabilitation programs in OLT candidates. These findings are somewhat surprising since the psychological burden on the OLT candidate is high (43), and the long waiting time and presence of symptoms related to liver cirrhosis possibly corrode compliance and motivation (34). Nonetheless, the dropout rate was low in all studies, and the most common reason for dropout was because patients were transplanted before the end of the study period.

This review has several limitations. First of all, this review was not pre-registered on the PROSPERO database, which could have caused reduced transparency of the applied search strategy of this review. Secondly, there are certain limitations regarding the studied evidence: most included studies consisted of small patient populations, and focused on different primary outcomes, which made the comparison and analysis of the studies challenging. In addition, most of the studies were non-randomized, which leads to a reduction in the analysis strength of this review. Finally, as the values of the baseline and post-training outcomes are not independent of each other, and correlations were not reported by the individual studies, meta-analyses were not possible (44). The high heterogeneity and lack of high-quality trials make it difficult to draw conclusions on the true effect of prehabilitation, when taking infrastructural differences, waiting time and clinical status as prognostic factors of success of the training programs in account. However, by strictly including only studies with patients having ESLD who are actively waitlisted for OLT, a bias of representing a “healthier” study group is prevented. Therefore, the strength of this review is, therefore, to represent the “most physically frail” patients, namely the OLT candidates with ESLD.

In our opinion, home-based training, which is supervised by a dedicated physical therapist and is combined with nutritional and educational support by a dietician, could be suitable for preoperative optimization until OLT. Patients might make some progress during these weeks of training, but, most importantly, deterioration of aerobic capacity could be prevented (27) and the number of hospital admissions due to decompensated liver disease during the waiting period could be reduced (45). To the best of our knowledge, the economic burden of the implementation of a prehabilitation program in this patient population has not been studied yet. One can imagine that the supervision and provision of a personalized training program for this frail population requires professional health-care workers as physiotherapists and dieticians. However, since previous studies showed cost-effectiveness for prehabilitation in patients undergoing abdominal surgery (46), we think that investing in personalized training programs for this specifically frail population could be beneficial. However, the effects of physical training in this patient population are still not decisive, and objectively measured effects of structured training programs on days of hospitalization, presence of complications and functional evolution after transplantation are scarce. Therefore, this review emphasizes the need for large (multicentre) longitudinal trials that not only study the physical effects, but also focus on possible improvement of surgical outcomes after a longer duration of training during the waiting period prior to OLT. Randomizing between training and no training is, in our opinion, not ethically justifiable, because various studies (45,47,48) have shown the benefits of improved physical capacity, activity, and muscle status with surgical outcome.

In conclusion, this systematic review found that prehabilitation in patients actively listed for OLT may improve aerobic and functional capacity, and, more importantly, that deterioration in aerobic and functional capacity could be countered by prehabilitation. Thereby, since no serious adverse events were reported and adherence to the training programs was high, we conclude that prehabilitation is safe and feasible in the OLT candidate. Thus, from our point of view, all patients awaiting OLT, especially the most physically frail ones, should be enrolled in predefined prehabilitation programs.

## Data Availability

The datasets used and/or analyzed during the current study are available from the corresponding author on reasonable request.

## Abbreviations

6MWD, six-minute walking distance; ERAS, enhanced recovery after surgery; ESLD, end-stage liver disease; F, delta point; GRADE, Grades of Recommendation, Assessment, Development and Evaluation for quality assessment of clinical studies; HR-QoL, health-related quality of life; i-CONTENT, international Consensus on Therapeutic Exercise aNd Training; ICU, intensive care unit; IQR, interquartile range; LFI, liver frailty index; MELD, model for end-stage liver disease; OLT, orthotopic liver transplantation; PRISMA, Preferred Reporting Items for Systematic Reviews and Meta-Analyses; PROSPERO, prospective register of systematic reviews; RCT, randomized controlled trial; SD, standard deviation; VO2 peak, oxygen consumption at peak exercise.
