# Supplementary material for: Physical Effects, Safety and Feasibility of Prehabilitation in Patients Awaiting Orthotopic Liver Transplantation, a Systematic Review
Source: Transpl Int. 2022 Sep 8;35:10330. doi: 10.3389/ti.2022.10330 (PMC9492850; doi:10.3389/ti.2022.10330)
Supplement: Supplementary file 1 [file DataSheet4.docx]

**Supplement 4. The i-CONTENT tool for assessing therapeutic quality of exercise programs employed in clinical trials** (14)

*Extracted from:*

Hoogeboom TJ, Kousemaker MC, van Meeteren NL, Howe T, Bo K, Tugwell P, Ferreira M, de Bie RA, van den Ende CH, Stevens-Lapsley JE. i-CONTENT tool for assessing therapeutic quality of exercise programs employed in randomised clinical trials. Br J Sports Med. 2020 Nov 3. doi: 10.1136

**Article 1.**

Limongi V, Dos Santos DC, Da Silva AMO, Ataide EC, Mei MFT, Udo EY, et al. Effects of a respiratory physiotherapeutic program in liver transplantation candidates. Transplant Proc. 2014;46(6):1775–7.

**i-CONTENT**

***1.*** *Patient selection:* The purpose of exercise does does not match patients’ problems, so HIGH risk of ineffectiveness.

***2.*** *Dosage of the exercise program:* The investigators did not apply a plausible or proven rationale based on anatomical, physiological, psychological, neurological, or behavioral relevance to the condition to determine the: frequency, intensity, and time of the exercise program matching the purpose of the exercise intervention, so HIGH risk of ineffectiveness.

***3.*** *Type of exercise:* The investigators did match the type of the exercise program with the purpose of the exercise therapy program, so LOW risk of ineffectiveness.

***4.*** *Qualified supervisor:* It can be assumed that the supervisor providing the program is inexperienced with the patient population or is insufficiently skilled to provide the exercise program, so HIGH risk of ineffectiveness.

***5.*** *Type and timing of outcome assessment:* The investigators used one or more performance-based outcome measures which reflect the goals and purpose of the exercise program to assess the effectiveness exercise therapy program. The measurements from the performance-based outcome measures have taken place within the time window where the expected effect would most likely take place, so LOW risk of ineffectiveness.

***6.*** *Safety of the exercise program:* The number and severity of the exercise-related adverse events in the study are not reported, so HIGH risk of ineffectiveness.

***7.*** *Adherence to the exercise program:* The level of adherence of patients to the exercise therapy program is not reported, so HIGH risk of ineffectiveness.

**Overall rating prehabilitation program:** HIGH risk of ineffectiveness

**Article 2.**

Debette-Gratien M, Tabouret T, Antonini M-T, Dalmay F, Carrier P, Legros R, et al. Personalized adapted physical activity before liver transplantation: acceptability and results. Transplantation. 2015 Jan;99(1):145–50.

**i-CONTENT**

***1.*** *Patient selection:* The purpose of exercise does match patients’ problems, so LOW risk of ineffectiveness.

***2.*** *Dosage of the exercise program:* The investigators applied a plausible or proven rationale based on anatomical, physiological, psychological, neurological, or behavioral relevance to the condition to determine the: frequency, intensity, and time of the exercise program matching the purpose of the exercise intervention., so LOW risk of ineffectiveness.

***3.*** *Type of exercise:* The investigators did match the type of the exercise program with the purpose of the exercise therapy program, so LOW risk of ineffectiveness.

***4.*** *Qualified supervisor:* It can be assumed that the supervisor providing the program is experienced with the patient population or is sufficiently skilled to provide the exercise program, so LOW risk of ineffectiveness.

***5.*** *Type and timing of outcome assessment:* The investigators used one or more performance-based outcome measures which reflect the goals and purpose of the exercise program to assess the effectiveness exercise therapy program. The measurements from the performance-based outcome measures have taken place within the time window where the expected effect would most likely take place, so LOW risk of ineffectiveness.

***6.*** *Safety of the exercise program:* The number and severity of the exercise-related adverse events in the study are in line with the expected number of adverse events for similar exercise program in similar populations, so LOW risk of ineffectiveness.

***7.*** *Adherence to the exercise program:* The level of adherence of patients to the exercise therapy program is not reported in data, so HIGH risk of ineffectiveness.

**Overall rating prehabilitation program:** LOW risk of ineffectiveness

**Article 3.**

Al-Judaibi B, Alqalami I, Sey M, Qumosani K, Howes N, Sinclair L, et al. Exercise training for liver transplant candidates. Transplant Proc. 2019;51(10):3330–7.

**i-CONTENT**

***1.*** *Patient selection:* The purpose of exercise does match patients’ problems, so LOW risk of ineffectiveness.

***2.*** *Dosage of the exercise program:* The investigators did not apply a plausible or proven rationale based on anatomical, physiological, psychological, neurological, or behavioral relevance to the condition to determine the: frequency, intensity, and time of the exercise program matching the purpose of the exercise intervention., so HIGH risk of ineffectiveness.

***3.*** *Type of exercise:* The investigators did not match the type of the exercise program with the purpose of the exercise therapy program, so HIGH risk of ineffectiveness.

***4.*** *Qualified supervisor:* It can be assumed that the supervisor providing the program is experienced with the patient population or is sufficiently skilled to provide the exercise program, so LOW risk of ineffectiveness.

***5.*** *Type and timing of outcome assessment:* The investigators did not use one or more performance-based outcome measures which reflect the goals and purpose of the exercise program to assess the effectiveness exercise therapy program, so HIGH risk of ineffectiveness.

***6.*** *Safety of the exercise program:* The number and severity of the exercise-related adverse events in the study are not reported, so HIGH risk of ineffectiveness.

***7.*** *Adherence to the exercise program:* The level of adherence of patients to the exercise therapy program is not reported, so HIGH risk of ineffectiveness.

**Overall rating prehabilitation program:** HIGH risk of ineffectiveness

**Article 4.**

Wallen MP, Keating SE, Hall A, Hickman IJ, Pavey TG, Woodward AJ, et al. Exercise training is safe and feasible in patients awaiting liver transplantation: A pilot randomized controlled trial. Vol. 25, Liver Transplantation. John Wiley and Sons Ltd; 2019. p. 1576–80.

**i-CONTENT**

***1.*** *Patient selection:* The purpose of exercise does match patients’ problems, so LOW risk of ineffectiveness.

***2.*** *Dosage of the exercise program:* The investigators applied a plausible or proven rationale based on anatomical, physiological, psychological, neurological, or behavioral relevance to the condition to determine the: frequency, intensity, and time of the exercise program matching the purpose of the exercise intervention., so LOW risk of ineffectiveness.

***3.*** *Type of exercise:* The investigators did match the type of the exercise program with the purpose of the exercise therapy program, so LOW risk of ineffectiveness

***4.*** *Qualified supervisor:* It can be assumed that the supervisor providing the program is experienced with the patient population or is sufficiently skilled to provide the exercise program, so LOW risk of ineffectiveness.

***5.*** *Type and timing of outcome assessment:* The investigators used one or more performance-based outcome measures which reflect the goals and purpose of the exercise program to assess the effectiveness exercise therapy program. The measurements from the performance-based outcome measures have taken place within the time window where the expected effect would most likely take place, so LOW risk of ineffectiveness.

***6.*** *Safety of the exercise program:* The number and severity of the exercise-related adverse events in the study are in line with the expected number of adverse events for similar exercise program in similar populations, so LOW risk of ineffectiveness

***7.*** *Adherence to the exercise program:* The level of adherence of patients to the exercise therapy program is deemed sufficient to assume that the proposed exercise therapy program was performed as originally intended, in terms of achieved exercise intensity, so LOW risk of ineffectiveness.

**Overall rating prehabilitation program:** LOW risk of ineffectiveness

**Article 5.**

Williams FR, Vallance A, Faulkner T, Towey J, Durman S, Kyte D, et al. Home-based exercise therapy in patients awaiting liver transplantation: A Feasibility Study. Liver Transplant. 2019;25(7):995–1006.

**i-CONTENT**

***1.*** *Patient selection:* The purpose of exercise does match patients’ problems, so LOW risk of ineffectiveness.

***2.*** *Dosage of the exercise program:* The investigators applied a plausible or proven rationale based on anatomical, physiological, psychological, neurological, or behavioral relevance to the condition to determine the: frequency, intensity, and time of the exercise program matching the purpose of the exercise intervention., so LOW risk of ineffectiveness.

***3.*** *Type of exercise:* The investigators did match the type of the exercise program with the purpose of the exercise therapy program, so LOW risk of ineffectiveness.

***4.*** *Qualified supervisor:* It can be assumed that the supervisor providing the program is experienced with the patient population or is sufficiently skilled to provide the exercise program, so LOW risk of ineffectiveness.

***5.*** *Type and timing of outcome assessment:* The investigators used one or more performance-based outcome measures which reflect the goals and purpose of the exercise program to assess the effectiveness exercise therapy program. The measurements from the performance-based outcome measures have taken place within the time window where the expected effect would most likely take place, so LOW risk of ineffectiveness.

***6.*** *Safety of the exercise program:* The number and severity of the exercise-related adverse events in the study are in line with the expected no of adverse events for similar exercise program in similar populations, so LOW risk of ineffectiveness.

***7.*** *Adherence to the exercise program:* The level of adherence of patients to the exercise therapy program is deemed sufficient to assume that the proposed exercise therapy program was performed as originally intended, in terms of achieved exercise intensity, so LOW risk of ineffectiveness.

**Overall rating prehabilitation program:** LOW risk of ineffectiveness

**Article 6.**

Morkane CM, Kearney O, Bruce DA, Melikian CN, Martin DS. An outpatient hospital-based exercise training program for patients with cirrhotic liver disease awaiting transplantation: A feasibility trial. Transplantation. 2019;104(1):97–103.

**i-CONTENT**

***1.*** *Patient selection:* The purpose of exercise does match patients’ problems, so LOW risk of ineffectiveness.

***2.*** *Dosage of the exercise program:* The investigators applied a plausible or proven rationale based on anatomical, physiological, psychological, neurological, or behavioral relevance to the condition to determine the: frequency, intensity, and time of the exercise program matching the purpose of the exercise intervention., so LOW risk of ineffectiveness.

***3.*** *Type of exercise:* The investigators did match the type of the exercise program with the purpose of the exercise therapy program, so LOW risk of ineffectiveness.

***4.*** *Qualified supervisor:* It can be assumed that the supervisor providing the program is experienced with the patient population or is sufficiently skilled to provide the exercise program, so LOW risk of ineffectiveness.

***5.*** *Type and timing of outcome assessment:* The investigators used one or more performance-based outcome measures which reflect the goals and purpose of the exercise program to assess the effectiveness exercise therapy program. The measurements from the performance-based outcome measures have taken place within the time window where the expected effect would most likely take place, so LOW risk of ineffectiveness.

***6.*** *Safety of the exercise program:* The number and severity of the exercise-related adverse events in the study are in line with the expected number of adverse events for similar exercise program in similar populations, so LOW risk of ineffectiveness.

***7.*** *Adherence to the exercise program:* The level of adherence of patients to the exercise therapy program is deemed sufficient to assume that the proposed exercise therapy program was performed as originally intended, in terms of achieved exercise intensity, so LOW risk of ineffectiveness.

**Overall rating prehabilitation program:** LOW risk of ineffectiveness

**Article 7.**

Chen HW, Ferrando A, White MG, Dennis RA, Xie J, Pauly M, et al. Home-based physical activity and diet intervention to improve physical function in advanced liver disease: A randomized pilot trial. Dig Dis Sci. 2020 Nov;65(11):3350–9.

**i-CONTENT**

***1.*** *Patient selection:* The purpose of exercise does match patients’ problems, so LOW risk of ineffectiveness.

***2.*** *Dosage of the exercise program:* The investigators applied a plausible or proven rationale based on anatomical, physiological, psychological, neurological, or behavioral relevance to the condition to determine the: frequency, intensity, and time of the exercise program matching the purpose of the exercise intervention., so LOW risk of ineffectiveness.

***3.*** *Type of exercise:* The investigators did match the type of the exercise program with the purpose of the exercise therapy program, so LOW risk of ineffectiveness.

***4.*** *Qualified supervisor:* It can be assumed that the supervisor providing the program is inexperienced with the patient population or is insufficiently skilled to provide the exercise program, so HIGH risk of ineffectiveness.

***5.*** *Type and timing of outcome assessment:* The investigators used one or more performance-based outcome measures which reflect the goals and purpose of the exercise program to assess the effectiveness exercise therapy program. The measurements from the performance-based outcome measures have taken place within the time window where the expected effect would most likely take place, so LOW risk of ineffectiveness

***6.*** *Safety of the exercise program:* The number and severity of the exercise-related adverse events in the study are not reported, so HIGH risk of ineffectiveness.

***7.*** *Adherence to the exercise program:* The level of adherence of patients to the exercise therapy program is not reported, so HIGH risk of ineffectiveness.

**Overall rating prehabilitation program:** HIGH risk of ineffectiveness

**Article 8.**

Lin F-P, Visina JM, Bloomer PM, Dunn MA, Josbeno DA, Zhang X, et al. Prehabilitation-driven changes in frailty metrics predict mortality in patients with advanced liver disease. Am J Gastroenterol. 2021 Oct;116(10):2105–17.

**i-CONTENT**

***1.*** *Patient selection:* The purpose of exercise does match patients’ problems, so LOW risk of ineffectiveness.

***2.*** *Dosage of the exercise program:* The investigators applied a plausible or proven rationale based on anatomical, physiological, psychological, neurological, or behavioral relevance to the condition to determine the: frequency, intensity, and time of the exercise program matching the purpose of the exercise intervention., so LOW risk of ineffectiveness.

***3.*** *Type of exercise:* The investigators did match the type of the exercise program with the purpose of the exercise therapy program, so LOW risk of ineffectiveness.

***4.*** *Qualified supervisor:* It can be assumed that the supervisor providing the program is inexperienced with the patient population or is insufficiently skilled to provide the exercise program, so HIGH risk of ineffectiveness.

***5.*** *Type and timing of outcome assessment:* The investigators used one or more performance-based outcome measures which reflect the goals and purpose of the exercise program to assess the effectiveness exercise therapy program. The measurements from the performance-based outcome measures have taken place within the time window where the expected effect would most likely take place, so LOW risk of ineffectiveness

***6.*** *Safety of the exercise program:* The number and severity of the exercise-related adverse events in the study are not reported, so HIGH risk of ineffectiveness.

***7.*** *Adherence to the exercise program:* The level of adherence of patients to the exercise therapy program is reported, so LOW risk of ineffectiveness.

**Overall rating prehabilitation program:** LOW risk of ineffectiveness
