# Supplementary material for: Physical Effects, Safety and Feasibility of Prehabilitation in Patients Awaiting Orthotopic Liver Transplantation, a Systematic Review
Source: Transpl Int. 2022 Sep 8;35:10330. doi: 10.3389/ti.2022.10330 (PMC9492850; doi:10.3389/ti.2022.10330)
Supplement: Supplementary file 2 [file DataSheet3.docx]

**Supplement 3. Quality of evidence of included articles rated according to GRADE** (17,18)**.**

*Extracted from:*

1. Guyatt GH, Oxman AD, Vist GE, Kunz R, Falck-Ytter Y, Alonso-Coello P, et al.

GRADE: an emerging consensus on rating quality of evidence and strength of recommendations. Bmj. 2008;336(7650):924-6.

2. Guyatt GH, Oxman AD, Sultan S, Glasziou P, Akl EA, Alonso-Coello, et al. GRADE Working Group. GRADE guidelines: 9. Rating up the quality of evidence. J Clin Epidemiol. 2011 Dec;64(12):1311-6.

**Article 1.**

Limongi V, Dos Santos DC, Da Silva AMO, Ataide EC, Mei MFT, Udo EY, et al. Effects of a respiratory physiotherapeutic program in liver transplantation candidates. Transplant Proc. 2014;46(6):1775–7.

**Down rating:**

*Risk of bias:* Moderate (no explanation how blinding was performed)

*Imprecision:* Yes (small study population)

*Inconsistency:* Yes (control and intervention group don’t have similar baseline characteristics)

*Indirectness:* Yes (conclusion is made on base of one significant outcome difference in lung function, however, other lung function characteristics did not differ between control and intervention group.

*Publication bias:* Possible (funding by sponsor)

**Up rating:**

*Large magnitude of effect:* Probably not

*Dose-response gradient:* Probably not

*All residual confounding would decrease magnitude of effect:* Possible

**Overall rating:** Low quality of evidence

**Article 2.**

Debette-Gratien M, Tabouret T, Antonini M-T, Dalmay F, Carrier P, Legros R, et al. Personalized adapted physical activity before liver transplantation: acceptability and results. Transplantation. 2015 Jan;99(1):145–50.

**Down rating:**

*Risk of bias:* High (no randomization, no blinding)

*Imprecision:* Yes (small study population)

*Inconsistency:* Probably not

*Indirectness:* Yes (strict inclusion criteria, so liver transplantation candidates who had comorbidities because of their liver disease were excluded (e.g. history of variceal bleeding).

Publication bias: Possible (no declaration section was reported in the article)

**Up rating:**

*Large magnitude of effect:* Probably not

*Dose-response gradient:* Probably not

*All residual confounding would decrease magnitude of effect:* Possible

**Overall rating:** Low quality of evidence

**Article 3.**

Al-Judaibi B, Alqalami I, Sey M, Qumosani K, Howes N, Sinclair L, et al. Exercise training for liver transplant candidates. Transplant Proc. 2019;51(10):3330–7.

**Down rating:**

*Risk of bias:* High (retrospective analysis)

*Imprecision:* Possible

*Inconsistency:* Yes (control and intervention group don’t have similar baseline characteristics)

*Indirectness:* Possible

*Publication bias:* Possible (no declaration section was reported in the article)

**Up rating:**

*Large magnitude of effect:* Probably not

*Dose-response gradient:* Probably not

*All residual confounding would decrease magnitude of effect:* Possible

**Overall rating:** Low quality of evidence

**Article 4.**

Wallen MP, Keating SE, Hall A, Hickman IJ, Pavey TG, Woodward AJ, et al. Exercise training is safe and feasible in patients awaiting liver transplantation: A pilot randomized controlled trial. Vol. 25, Liver Transplantation. John Wiley and Sons Ltd; 2019. p. 1576–80.

**Down rating:**

*Risk of bias:* Low (randomization process is well documented)

*Imprecision:* Possible (small study population, > 40% of eligible study participants declined)

*Inconsistency:* Probably not

*Indirectness:* Probably not

*Publication bias:* Possible (no declaration section was reported in the article)

**Up rating:**

*Large magnitude of effect:* Possible

*Dose-response gradient:* Probably not

*All residual confounding would decrease magnitude of effect:* Possible

**Overall rating:** Moderate quality of evidence

**Article 5.**

Williams FR, Vallance A, Faulkner T, Towey J, Durman S, Kyte D, et al. Home-based exercise therapy in patients awaiting liver transplantation: A Feasibility Study. Liver Transplant. 2019;25(7):995–1006.

**Down rating:**

*Risk of bias:* High (no randomization, no blinding)

*Imprecision:* Possible (small study population)

*Inconsistency:* Probably not

*Indirectness:* Possible (strict inclusion criteria)

*Publication bias:* Probably not (funding from own university)

**Up rating:**

*Large magnitude of effect*: Probably not

*Dose-response gradient:* Probably not

*All residual confounding would decrease magnitude of effect:* Possible

**Overall rating:** Low quality of evidence

**Article 6.**

Morkane CM, Kearney O, Bruce DA, Melikian CN, Martin DS. An outpatient hospital-based exercise training program for patients with cirrhotic liver disease awaiting transplantation: A feasibility trial. Transplantation. 2019;104(1):97–103.

**Down rating:**

*Risk of bias:* Moderate (prospective study with matched control group, but no randomization and blinding)

*Imprecision:* Possible (small study population, study completion of 50% of participants)

*Inconsistency:* Probably not

*Indirectness:* Probably not (all included patients were listed for liver transplantation)

*Publication bias:* Possible (no declaration section was reported in the article)

**Up rating:**

*Large magnitude of effect:* Probably not

*Dose-response gradient:* Probably not

*All residual confounding would decrease magnitude of effect:* Probably not.

**Overall rating:** Low quality of evidence

**Article 7.**

Chen HW, Ferrando A, White MG, Dennis RA, Xie J, Pauly M, et al. Home-based physical activity and diet intervention to improve physical function in advanced liver disease: A randomized pilot trial. Dig Dis Sci. 2020 Nov;65(11):3350–9.

**Down rating:**

*Risk of bias:* Low (patient blinding and randomization was executed)

*Imprecision:* Possible (small study population)

*Inconsistency:* Probably not

*Indirectness:* Possible (9% of eligible candidates gave informed consent for the study)

*Publication bias:* Probably not (sponsor was blinded to intervention, funding received from own university)

**Up rating:**

*Large magnitude of effect:* Probably not

*Dose-response gradient:* Probably not.

*All residual confounding would decrease magnitude of effect:* Probably not.

**Overall rating:** Low quality of evidence

**Article 8.**

Lin F-P, Visina JM, Bloomer PM, Dunn MA, Josbeno DA, Zhang X, et al. Prehabilitation-driven changes in frailty metrics predict mortality in patients with advanced liver disease. Am J Gastroenterol. 2021 Oct;116(10):2105–17.

**Down rating:**

*Risk of bias:* Yes (no randomization)

*Imprecision:* No (large study population)

*Inconsistency:* Probably not.

*Indirectness:* Probably not.

*Publication bias:* Probably not (although it was funded by an external party, the funding was received from the Spanish Association for the Study of the Liver (AEEH).

**Up rating:**

*Large magnitude of effect:* Possible

*Dose-response gradient:* Probably not

*All residual confounding would decrease magnitude of effect:* Probably not.

**Overall rating:** Moderate quality of evidence
