# Supplementary material for: Physical Effects, Safety and Feasibility of Prehabilitation in Patients Awaiting Orthotopic Liver Transplantation, a Systematic Review
Source: Transpl Int. 2022 Sep 8;35:10330. doi: 10.3389/ti.2022.10330 (PMC9492850; doi:10.3389/ti.2022.10330)
Supplement: Supplementary file 3 [file DataSheet2.docx]

**Supplement 2. Literature database search strategy.**

Literature database search strategy by W. Bramer, PhD, information specialist at Erasmus University Medical Center, Rotterdam, The Netherlands.

**Date of search:** December 21st, 2021

| **Database searched** | **via** | **Years of coverage** | **Records** | **Records after duplicates removed** |
| --- | --- | --- | --- | --- |
| Embase | Embase.com | 1971 - 2021 | 449 | 427 |
| Medline ALL | Ovid | 1946 - 2021 | 150 | 23 |
| Web of Science Core Collection* | Web of Knowledge | 1975 - 2021 | 158 | 30 |
| Cochrane Central Register of Controlled Trials | Wiley | 1992 - 2021 | 35 | 20 |
| Other sources: Google Scholar (100 top-ranked) | | | 100 | 63 |
| **Total** | | | **892** | **563** |

*Science Citation Index Expanded (1975-2021) ; Social Sciences Citation Index (1975-2021) ; Arts & Humanities Citation Index (1975-2021) ; Conference Proceedings Citation Index- Science (1990-2021) ; Conference Proceedings Citation Index- Social Science & Humanities (1990-2021) ; Emerging Sources Citation Index (2015-2021)

**embase.com**

('liver surgery'/exp OR 'liver cancer'/exp/dm_su OR (((liver OR hepat*) NEAR/3 (transplant* OR allotransplant* OR homotransplant* OR graft* OR allograft* OR homograft* OR surg* OR operat* OR resection*)) OR hepatectom*):ab,ti,kw) AND ((('preoperative period'/de OR 'preoperative evaluation'/de OR 'preoperative care'/de) AND ('rehabilitation'/de OR 'kinesiotherapy'/exp OR 'oxygen consumption'/exp OR 'aerobic capacity'/de OR 'exercise test'/de OR 'exercise'/de OR 'exercise tolerance'/de OR 'rehabilitation care'/de OR 'cardiovascular system examination'/de OR 'physiotherapy'/de)) OR (prehabilitation* OR ((pre-operat* OR preoperat* OR waiting OR waitlist OR wait-list OR candidate*) NEAR/6 (kinesiotherap* OR exercise* OR aerobic-capacit* OR oxygen-uptake* OR oxygen-consum* OR optimizat* OR optimisat* OR rehabilitat* OR cardiac-evaluat* OR cardiac-assess* OR cardiac-investigat* OR cardiovascular*-evaluat* OR cardiovascular*-assess* OR cardiovascular*-investigat* OR physical-therap* OR physiotherap*))):Ab,ti,kw)

**Medline ALL Ovid**

(Liver/su OR Liver Transplantation/ OR Liver Neoplasms/su OR (((liver OR hepat*) ADJ3 (transplant* OR allotransplant* OR homotransplant* OR graft* OR allograft* OR homograft* OR surg* OR operat* OR resection*)) OR hepatectom*).ab,ti,kf.) AND (((Preoperative Period/ OR Preoperative Care/ ) AND (Rehabilitation/ OR Exercise Therapy/ OR Oxygen Consumption/ OR Exercise Test/ OR Exercise/ OR Exercise Tolerance/ OR exp Physical Therapy Modalities/)) OR (prehabilitation* OR ((pre-operat* OR preoperat* OR waiting OR waitlist OR wait-list OR candidate*) ADJ6 (kinesiotherap* OR exercise* OR aerobic-capacit* OR oxygen-uptake* OR oxygen-consum* OR optimizat* OR optimisat* OR rehabilitat* OR cardiac-evaluat* OR cardiac-assess* OR cardiac-investigat* OR cardiovascular*-evaluat* OR cardiovascular*-assess* OR cardiovascular*-investigat* OR physical-therap* OR physiotherap*))).ab,ti,kf.)

**Web of Science Core Collection**

TS=(((((liver OR hepat*) NEAR/2 (transplant* OR allotransplant* OR homotransplant* OR graft* OR allograft* OR homograft* OR surg* OR operat* OR resection*)) OR hepatectom*)) AND ((prehabilitation* OR ((pre-operat* OR preoperat* OR waiting OR waitlist OR wait-list OR candidate*) NEAR/5 (kinesiotherap* OR exercise* OR aerobic-capacit* OR oxygen-uptake* OR oxygen-consum* OR optimizat* OR optimisat* OR rehabilitat* OR cardiac-evaluat* OR cardiac-assess* OR cardiac-investigat* OR cardiovascular*-evaluat* OR cardiovascular*-assess* OR cardiovascular*-investigat* OR physical-therap* OR physiotherap*)))))

**Cochrane CENTRAL register of trials**

((((liver OR hepat*) NEAR/3 (transplant* OR allotransplant* OR homotransplant* OR graft* OR allograft* OR homograft* OR surg* OR operat* OR resection*)) OR hepatectom*):ab,ti,kw) AND ((prehabilitation* OR ((pre NEXT operat* OR preoperat* OR waiting OR waitlist OR wait NEXT list OR candidate*) NEAR/6 (kinesiotherap* OR exercise* OR aerobic NEXT capacit* OR oxygen NEXT uptake* OR oxygen NEXT consum* OR optimizat* OR optimisat* OR rehabilitat* OR cardiac NEXT evaluat* OR cardiac NEXT assess* OR cardiac NEXT investigat* OR cardiovascular* NEXT evaluat* OR cardiovascular* NEXT assess* OR cardiovascular* NEXT investigat* OR physical NEXT therap* OR physiotherap*))):Ab,ti,kw)

**Google Scholar**

"liver|hepatic transplant|allotransplant|homotransplant|graft|allograft|homograft|resection" prehabilitation|"pre-operative|preoperative kinesiotherapy|exercise|aerobic-capacity|oxygen-uptake|oxygen-consumption|physical-therapy|physiotherapy"

'liver|hepatic transplant|allotransplant|homotransplant|graft|allograft|homograft|resection' prehabilitation|'pre-operative|preoperative kinesiotherapy|exercise|aerobic-capacity|oxygen-uptake|oxygen-consumption|physical-therapy|physiotherapy'
